# Flexural Strength Design of Hybrid FRP-Steel Reinforced Concrete Beams

**DOI:** 10.3390/ma14216400

**Published:** 2021-10-25

**Authors:** Binbin Zhou, Ruo-Yang Wu, Yangqing Liu, Xiaohui Zhang, Shiping Yin

**Affiliations:** 1 Jiangsu Key Laboratory of Environmental Impact and Structural Safety in Engineering, School of Mechanics & Civil Engineering, China University of Mining and Technology, Xuzhou 221116, China; 2 State Key Laboratory for Geomechanics & Deep Underground Engineering, China University of Mining and Technology, Xuzhou 221116, China; 3 Wilson and Company, South Jordan, UT 84096, USA; rywuandrew@gmail.com; 4 School of Civil Engineering, Chongqing Jiaotong University, Chongqing 400074, China; 5 Key Laboratory of C & PC Structures of Ministry of Education, National Prestress Engineering Research Center, Southeast University, Nanjing 210096, China; zhangxiaohui@seu.edu.cn

**Keywords:** hybrid reinforced concrete beams, flexural failure modes, nominal flexural strength, strength reduction factor, ductility analysis

## Abstract

Through proper arranging of a hybrid combination of longitudinal fiber reinforced polymer (FRP) bars and steel bars in the tensile region of the beam, the advantages of both FRP and steel materials can be sufficiently exploited to enhance the flexural capacity and ductility of a concrete beam. In this paper, a methodology for the flexural strength design of hybrid FRP-steel reinforced concrete (RC) beams is proposed. Firstly, based on the mechanical features of reinforcement and concrete and according to the latest codified provisions of longitudinal reinforcement conditions to ensure ductility level, the design-oriented allowable ranges of reinforcement ratio corresponding to three common flexural failure modes are specified. Subsequently, the calculation approach of nominal flexural strength of hybrid FRP-steel RC beams is established following the fundamental principles of equilibrium and compatibility. In addition to the common moderately-reinforced beams, the proposed general calculation approach is also applicable to lightly-reinforced beams and heavily-reinforced beams, which are widely used but rarely studied. Furthermore, the calculation process is properly simplified and the calculation accuracy is validated by the experimental results of hybrid FRP-steel RC beams in the literature. Finally, with the ductility analysis, a novel strength reduction factor represented by net tensile steel strain and reinforcement ratio is proposed for hybrid FRP-steel RC beams.

## 1. Introduction

The use of fiber reinforced polymer (FRP) as longitudinal reinforcement of concrete members has gained popularity due to its advantages, such as high strength, light weight, and non-corrosive properties. However, the unfavorable structural performance of pure FRP reinforced concrete (RC) beams, such as wide crack width and large deflection at service stage, and low ductility (deformability) at the ultimate stage, restricts the application of FRP reinforcement. To address the aforementioned issues, hybrid combination of FRP and steel longitudinal reinforcement in the tensile zone of a concrete beam has been proposed [1,2,3]. In combination, the beneficial properties of both FRP and steel materials could be efficiently exploited. Specifically, the FRP reinforcement near the outer surface of the tensile zone provides high strength and durability, and the steel reinforcement at the inner level of the tensile zone improves serviceability and ductility [2,3,4].

In recent years, extensive research has been performed by kinds of measures on flexural performance of hybrid FRP-steel reinforced concrete beams [1,2,4,5,6,7,8,9,10,11,12,13,14,15,16,17,18,19,20,21,22,23,24]. Aiello et al. [1] investigated the serviceability and bearing capacity of hybrid aramid fiber reinforced polymer (AFRP)-steel RC beams by experiments, and verified the beneficial effect of adding steel reinforcement on reducing crack width and deflection at service stage and improving ductility at the ultimate stage. Additionally, the moment-curvature relationship incorporating a tension stiffening effect was derived to predict the overall flexural behavior of hybrid RC beams. Leung et al. [2] analytically derived the balanced reinforcement ratios corresponding to possible flexural failure modes, and then carried out experiments to validate the theoretical analysis. Qu et al. [5] experimentally confirmed that adding an adequate amount of steel reinforcement to glass fiber reinforced polymer (GFRP) RC beams could effectively optimize structural performance. The effective reinforcement ratio was proposed to analytically predict the possible flexural failure modes of hybrid GFRP-steel RC beams. Furthermore, the calculation model of flexural strength of hybrid RC beams with the preferred flexural failure mode (steel yielding followed by concrete crushing) was proposed. The flexural strength and deflection of high-strength concrete beams reinforced with multiple layers of reinforcement and combinations of different reinforcement types (steel, GFRP, and carbon FRP (CFRP) bars) were experimentally and analytically evaluated by Yoon et al. [6]. The test results showed that the low post cracking stiffness, high deflection, deep crack propagation, large crack width, and low ductility of FRP bar reinforced beams were controlled and improved by hybrid reinforcing with steel bars. Safan [7] proposed and experimentally validated a comprehensive analytical model to appraise the flexural capacity of hybrid GFRP-steel RC beams with a favorable ductile manner. Moreover, a minimum limit for the tensile strain of steel reinforcement was specified to guarantee the ductile failure of hybrid RC beams. Liu et al. [8] experimentally investigated the flexural strength, deflection, and crack behavior of high strength concrete beams reinforced with hybrid GFRP-steel reinforcement to verify the influence of arrangement of rebar layers and support design of hybrid reinforced concrete structures. Additionally, theoretical models were proposed to predict load-carrying capacity and failure modes. Ge et al. [9] experimentally investigated the flexural behavior of hybrid basalt fiber reinforced polymer (BFRP)-steel RC beams and found that the ratio of BFRP to steel reinforcement was critical to the ductility of hybrid RC beams. In addition, the occurrence criteria and flexural capacity of hybrid RC beams with the desirable flexural failure mode were analytically specified. El Refai et al. [10] experimentally studied the structural performance of concrete beams reinforced with a combination of steel and GFRP bars. The load-carrying capacity, deflection, crack widths, and deformability of hybrid-reinforced concrete beams were predicted by the presented models. Based on the conventional sectional analysis, Kara et al. [11] proposed the advanced moment-curvature relationship considering tension stiffening effect and numerically predicted the flexural capacity and the ultimate displacement of hybrid FRP-steel RC beams characterized by the possible flexural failure modes. Zhou et al. [12] presented the general numerical model to calculate the complete deformation development process of hybrid FRP-steel RC beams. It is applicable to beams with common flexural failure modes since the tension stiffening effect in the tensile region and softening behavior of compressive concrete are both considered. Bencardino et al. [13] carried out numerical analysis on hybrid FRP-steel RC beams using a simple and reliable two-dimensional finite element (FE) model. The tension stiffening effect was simulated by defining the post-crack behavior of tensile concrete. To precisely simulate the load-carrying capacity and deformation development of concrete beams reinforced with a hybrid combination of AFRP and steel bars, Hawileh et al. [14] developed a three-dimensional FE model, in which the constituent material nonlinearities and bond performance between the reinforcing bars and surrounding concrete were incorporated. Gu et al. [15] performed bond tests and four-point bending tests to determine the effect of bonding performance of GFRP rebars on the flexural behavior of hybrid GFRP-steel RC beams and compared their flexural behavior. Subsequently, the bond-slip relationship between GFRP and concrete, obtained from pull-out tests, was implemented into an FE model to simulate the flexural behavior of the beams. Araba et al. [16] experimentally and analytically studied the structural performance of continuous hybrid GFRP-steel RC beams. The results illustrated that the hybrid RC beams could obtain the desirable ductility and moment redistribution by adopting the proper ratio of GFRP to steel reinforcement. Pang et al. [17] analytically derived the proper reinforcement ratio limits to ensure the ductile failure of hybrid GFRP-steel RC beams and predicted the flexural strength. In addition, a new ductility index comprehensively considering the factors of deformability and energy absorption capacity was defined according to the combination and mechanical properties of GFRP and steel reinforcement. Furthermore, the effects of various parameters on ductility were discussed. Linh et al. [18] numerically investigated the mechanical performance and ductility of hybrid RC beams. The effects of ratio of FRP to steel reinforcement, location of FRP reinforcement, type of FRP reinforcement, and concrete compressive strength, on the flexural performance of the beams, were parametrically investigated. Nguyen et al. [19] experimentally studied the flexural behavior of hybrid GFRP-steel RC beams including response stages, failure modes, crack patterns, stiffness, toughness and ductility. It indicated that the effects of reinforcement configuration and ratio of GFRP to steel reinforcement on the crack patterns, stiffness, ductility and toughness of hybrid RC beams are significant. In addition, Nguyen et al. [20] proposed a theoretical approach to estimate the minimum and maximum reinforcement ratios for hybrid RC beams.

Existing studies [5,9,10,12,15,16,17,21,22,23,24,25,26] indicated that hybrid FRP-steel RC beams exhibit the following three flexural failure modes related to the different FRP and steel reinforcement ratios:

- Failure mode I: FRP reinforcement ruptures after tensile steel reinforcement yielding without concrete crushing (lightly reinforced beam);
- Failure mode II: concrete crushing occurs after tensile steel reinforcement yielding without rupture of FRP reinforcement (moderately reinforced beam);
- Failure mode III: concrete crushing occurs while tensile steel and FRP reinforcement are in the elastic state (heavily reinforced beam).

Failure mode II is preferable among the three modes since it is more progressive and has superior ductility or deformability to the others. In practical design, it is recommended to use “ductile beam” to be economical with design simplicity [3,27]. Specifically, the strength properties of materials, such as FRP, steel, and concrete, can be fully exploited. Therefore, most theoretical models [1,2,5,6,7,8,9,10,15,16,17,26] have focused on the evaluation of flexural capacity of hybrid FRP-steel RC beams featured by failure mode II. Rare calculation models are applicable to beams characterized by failure modes I and III, which are also quite common in some practical design and application cases; for example, failure mode I has often occurred in bridge girders and deck slabs [28]. On the other hand, the available studies [29,30,31,32] have presented design strategies regarding the section strength of RC flexural members retrofitted with externally bonded FRP reinforcement according to failure modes. Furthermore, the ACI 440.2R-17 [33] has defined the strength reduction factor (*ϕ*) considering different ductility and safety levels, which is critical for the design and application of retrofitted RC flexural members. However, the relevant provisions are almost identical to those of ACI 318-19 [34] and ACI 318-14 [35], served for steel RC members; the influence of the externally bonded FRP reinforcement on ductility of members is not sufficiently reflected, especially for lightly reinforced members, which are not defined in the mentioned codes. Currently, due to a lack of evaluation of the ductility level of hybrid FRP-steel RC beams, which can be considered as a special type of retrofitted RC flexural member with FRP reinforcement, the proper suggestions about strength reduction factor are not proposed and the corresponding design strategies need to be specified.

To deal with the problems mentioned above, a comprehensive methodology for the flexural strength design of hybrid FRP-steel reinforced concrete beams is proposed in this paper based on the design philosophy and provisions of the relevant ACI codes. Specifically, the design-oriented allowable ranges of reinforcement ratio, corresponding to three common flexural failure modes, are specified by considering the mechanical features of reinforcement and concrete and according to the latest codified provisions of longitudinal reinforcement conditions in the ACI 318-19. Moreover, a thorough analytical approach is presented to evaluate the flexural strength of hybrid FRP-steel RC beams with common flexural failure modes. The approach is established following the fundamental principles of equilibrium and compatibility and is applicable to both singly- and doubly-reinforced concrete beams. The calculation of flexural strength of lightly-reinforced concrete beams is significantly simplified using the presented relationship between reinforcement ratio and relative neutral axis depth. Subsequently, the proposed analytical approach is validated by the experimental results of hybrid FRP-steel RC beams available in the literature [1,2,5,7,16,21,23]. Finally, a novel strength reduction factor represented by net tensile steel strain and reinforcement ratio is proposed for hybrid FRP-steel RC beams with the common flexural failure modes through ductility analysis.

## 2. Division of Flexural Failure Modes

Considering the balanced failure states of hybrid FRP-steel RC beams, the approximate ranges of reinforcement ratio to discern three common flexural failure modes were established by a number of studies [5,6,7,8,9,10,11,14,15,16,17,21,22,23,24,25,26]. Based on these, and according to the latest codified provisions of longitudinal reinforcement conditions to guarantee the sufficient ductility level [34,36], the design-oriented allowable ranges of reinforcement ratio corresponding to common flexural failure modes are specified as follows.

### 2.1. Primary Hypotheses

Following the design philosophy and definitions of the relevant ACI codes [3,27,34,35,36], the following primary hypotheses are made to implement the following sectional analysis:

1. Strain varies linearly through the cross-section (that is, plane sections remain plane);
2. Perfect bond exists between steel and FRP reinforcement and concrete;
3. Concrete strain in compression is limited to 0.003, and under this condition the Whitney equivalent stress block is a valid substitution for nonlinear stress distribution;
4. Stress-strain response of FRP reinforcement is linear-elastic up to failure;
5. Steel reinforcement performs the ideal bilinear elastic-plastic behavior;
6. Tensile strength of concrete is ignored.

**Figure 1:**
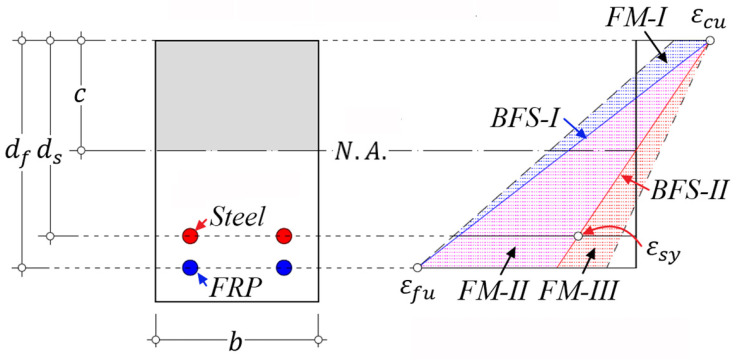
Features of strain distribution on a cross-section of hybrid RC beam at the ultimate state. (Note: N.A. = neutral axis.).

Corresponding to the aforementioned three types of flexural failure modes, the features of strain distribution on a cross-section of hybrid RC beam at the ultimate state could be schematically illustrated by Figure 1, where the letters FM and BFS stand for failure mode and balanced failure state, respectively, and the number next to the letters represents the specific category of failure mode and state defined in this study, respectively. The details are introduced as follows.

### 2.2. Failure Mode I (FM-I)

FRP reinforcement rupture is induced at $\varepsilon_{f}=\varepsilon_{fu}$; yielding of steel reinforcement occurs $\varepsilon_{s}>\varepsilon_{sy}$; and concrete is not crushed $\varepsilon_{c}<\varepsilon_{cu}$. (Note: $\varepsilon_{f}$ is the tensile strain of FRP; $\varepsilon_{fu}$ is the ultimate tensile strain of FRP; $\varepsilon_{s}$ is the tensile strain of steel; $\varepsilon_{sy}$ is the yield strain of steel; $\varepsilon_{c}$ is the strain at the extreme compressive fiber of concrete; and $\varepsilon_{cu}$ is the ultimate strain of compressive concrete of 0.003 [3,27,34,35,36].)

Hybrid RC beam with failure mode I is lightly reinforced with FRP and steel bars and is governed by the tensile failure of FRP reinforcement. As the reinforcement ratio increases, the contribution of compressive concrete to flexural capacity and deformation gradually increases [12]. The balanced failure state I (BFS-I) shown in Figure 1 is defined as the state with the simultaneous occurrence of concrete crushing and FRP rupture. According to the force equilibrium and strain compatibility, Equations (1) and (2) [27,34,35] are presented:

(1) $$f_{fu}A_{f}+f_{sy}A_{s}=0.85\beta_{1}f_{c}^{\prime }bd_{f}\frac{\varepsilon_{cu}}{\varepsilon_{cu}+\varepsilon_{fu}}$$

(2) $$\beta_{1}=0.85-0.05\frac{f_{c}^{\prime }-27.6}{6.9};0.65\leq \beta_{1}\leq 0.85$$

where $f_{fu}$ is the tensile strength of FRP reinforcement;$\;f_{sy}$ is the yield strength of steel reinforcement; $A_{f}$ and $A_{s}$ are the cross-sectional areas of FRP and steel reinforcement, respectively;$\;\beta_{1}$ is the parameter relating depth of the Whitney equivalent stress block to neutral axis depth [27,34,35]; $f_{c}^{\prime }$ is the compressive strength of concrete; b is the width of beam section; and $d_{f}$ is the depth of FRP reinforcement on beam section measured from the extreme compression fiber.

Considering $\eta =d_{s}/d_{f}$, $\rho_{s}=A_{s}/\left(bd_{s}\right)$, and $\rho_{f}=A_{f}/\left(bd_{f}\right)$, Equation (1) can be rearranged as:

(3) $$\rho_{f}+\rho_{s}\eta \frac{f_{sy}}{f_{fu}}=0.85\beta_{1}\frac{f_{c}^{\prime }}{f_{fu}}\frac{\varepsilon_{cu}}{\varepsilon_{cu}+\varepsilon_{fu}}$$

where $d_{s}$ is the depth of steel reinforcement on beam section measured from the extreme compression fiber; η is the geometrical parameter; and $\rho_{s}$ and $\rho_{f}$ are the steel and FRP reinforcement ratios, respectively.

The left side of Equation (3) is defined as the mechanical reinforcing index [17], $\rho_{l}^{com}$, as shown in Equation (4):

(4) $$\rho_{l}^{com}=\rho_{f}+\rho_{s}\eta \frac{f_{sy}}{f_{fu}}$$

The right side of Equation (3) is defined as the balanced reinforcement ratio [17], $\rho_{l,b}^{com}$, as shown in Equation (5):

(5) $$\rho_{l,b}^{com}=0.85\beta_{1}\frac{f_{c}^{\prime }}{f_{fu}}\frac{\varepsilon_{cu}}{\varepsilon_{cu}+\varepsilon_{fu}}$$

For hybrid RC beam with failure mode I, its mechanical reinforcing index $\rho_{l}^{com}$ should satisfy the condition of $\rho_{l}^{com}<$ $\rho_{l,b}^{com}$. Furthermore, $\rho_{l}^{com}$ should be not less than the minimum FRP reinforcement ratio $\rho_{f,min}=0.41\sqrt{f_{c}^{\prime }}/f_{fu}$ defined by the ACI 440.1R-15 [3,36], that is, $\rho_{l}^{com}\geq \rho_{f,min}$. Hence, the allowable range of mechanical reinforcing index corresponding to failure mode I is expressed by $\rho_{f,min}\leq \rho_{l}^{com}<$ $\rho_{l,b}^{com}$.

### 2.3. Failure Mode II (FM-II)

FRP reinforcement is in the elastic state $\varepsilon_{f}<\varepsilon_{fu}$; yielding of steel reinforcement is induced $\varepsilon_{s}\geq \varepsilon_{sy}$; and concrete crushes at $\varepsilon_{c}=\varepsilon_{cu}$.

Hybrid RC beam with failure mode II is moderately reinforced with FRP and steel bars. The flexural failure will be initiated with steel yielding and then followed by concrete crushing, which is the governing factor. According to the equilibrium and compatibility conditions on cross-section, Equations (6) and (7) are established:

(6) $$f_{sy}A_{s}+f_{f}A_{f}=0.85\beta_{1}f_{c}^{\prime }bd_{s}\frac{\varepsilon_{cu}}{\varepsilon_{cu}+\varepsilon_{st}}$$

(7) $$f_{f}=E_{f}\varepsilon_{cu}\left(\frac{1+\varepsilon_{st}/\varepsilon_{cu}}{\eta }-1\right)$$

where $\varepsilon_{st}$ is the net tensile strain in the extreme tensile layer of steel reinforcement ($\varepsilon_{st}\geq \varepsilon_{sy}$);$f_{f}$ is the tensile stress of FRP reinforcement; and $E_{f}$ is the elastic modulus of FRP reinforcement.

Based on the presented Equations (6) and (7), the limitations of steel-FRP reinforcement ratio can be derived from the net tensile steel strain level to satisfy the requirement of sectional ductility defined in the ACI Codes (ACI 318-19 [34] and ACI 318-14 [35]) for steel RC flexural members. The relationship between net tensile steel strain and reinforcement ratios is expressed in Equations (8) and (9):

(8) $$\rho_{s}+\rho_{f}\frac{E_{f}}{E_{s}}\frac{\left(\frac{1+\varepsilon_{st}/\varepsilon_{cu}}{\eta }-1\right)}{\mu \eta }=\frac{0.85\beta_{1}f_{c}^{\prime }}{\left(1+\varepsilon_{st}/\varepsilon_{cu}\right)f_{sy}}$$

(9) $$\mu =\frac{\varepsilon_{sy}}{\varepsilon_{cu}}$$

where μ is a material parameter; and $E_{s}$ is the elastic modulus of steel reinforcement.

The left side of Equation (8) is defined as the effective reinforcement stiffness [17] $\rho_{\varepsilon_{st}}^{com}$ in Equation (10):

(10) $$\rho_{\varepsilon_{st}}^{com}=\rho_{s}+\rho_{f}\frac{E_{f}}{E_{s}}\frac{\left(\frac{1+\varepsilon_{st}/\varepsilon_{cu}}{\eta }-1\right)}{\mu \eta }$$

The right side of Equation (8) is defined as the balanced reinforcement ratio [17] $\rho_{\varepsilon_{st},b}^{com}$ at the net tensile steel strain level of $\varepsilon_{st}$, shown in Equation (11):

(11) $$\rho_{\varepsilon_{st},b}^{com}=\frac{0.85\beta_{1}f_{c}^{\prime }}{\left(1+\varepsilon_{st}/\varepsilon_{cu}\right)f_{sy}}$$

It is noted that the comparison between effective reinforcement stiffness $\rho_{\varepsilon_{st}}^{com}$ and the balanced reinforcement ratio $\rho_{\varepsilon_{st},b}^{com}$ should be performed at the identical net tensile steel strain level of $\varepsilon_{st}$.

At the critical state of failure mode II, that is, the simultaneous occurrence of concrete crushing and steel yielding, defined as the balanced failure state II (BFS-II) and shown in Figure 1, the effective reinforcement stiffness $\rho_{\varepsilon_{sy}}^{com}$ satisfies the condition presented in Equation (12):

(12) $$\rho_{\varepsilon_{sy}}^{com}=\rho_{s}+\rho_{f}\frac{E_{f}}{E_{s}}\frac{\left(\frac{1+\mu }{\eta }-1\right)}{\mu \eta }=\rho_{\varepsilon_{sy},b}^{com}=\frac{0.85\beta_{1}f_{c}^{\prime }}{\left(1+\mu \right)f_{sy}}$$

Hence, for the hybrid RC beams with failure mode II, its mechanical reinforcing index $\rho_{l}^{com}$ should satisfy the condition of $\rho_{l}^{com}>$ $\rho_{l,b}^{com}$ and the effective reinforcement stiffness $\rho_{\varepsilon_{sy}}^{com}$ should satisfy the condition of $\rho_{\varepsilon_{sy}}^{com}\leq$ $\rho_{\varepsilon_{sy},b}^{com}$.

To ensure the ductile behavior of steel RC beams, the latest ACI 318-19 suggested that the net tensile steel strain should be at least $\varepsilon_{sy}+0.003$ [34]. Correspondingly, in terms of hybrid RC beam, the allowable effective reinforcement stiffness $\rho_{\varepsilon_{sy+0.003}}^{com}$ should satisfy the condition expressed by Equation (13):

(13) $$\rho_{\varepsilon_{sy+0.003}}^{com}\leq \rho_{\varepsilon_{sy+0.003,b}}^{com}=\frac{0.85\beta_{1}f_{c}^{\prime }}{\left(1+\left(\varepsilon_{sy}+0.003\right)/\varepsilon_{cu}\right)f_{sy}}=\frac{0.85\beta_{1}f_{c}^{\prime }}{\left(2+\mu \right)f_{sy}}$$

where $\rho_{\varepsilon_{sy+0.003,b}}^{com}$ is the balanced reinforcement ratio at the level of net tensile steel strain being equal to $\varepsilon_{sy}+0.003$.

### 2.4. Failure Mode III (FM-III)

In this failure mode, both FRP reinforcement and steel reinforcement are in the elastic state: $\varepsilon_{f}<\varepsilon_{fu}$ and $\varepsilon_{s}<\varepsilon_{sy}$; and concrete crushing occurs at $\varepsilon_{c}=\varepsilon_{cu}$.

The flexural failure of the hybrid RC beam, heavily-reinforced with FRP and steel bars, is governed by concrete crushing, and the strength properties of steel and FRP reinforcement are not sufficiently exploited. Its effective reinforcement stiffness $\rho_{\varepsilon_{sy}}^{com}$ satisfies the condition of $\rho_{\varepsilon_{sy}}^{com}>$ $\rho_{\varepsilon_{sy},b}^{com}$.

**Figure 2:**
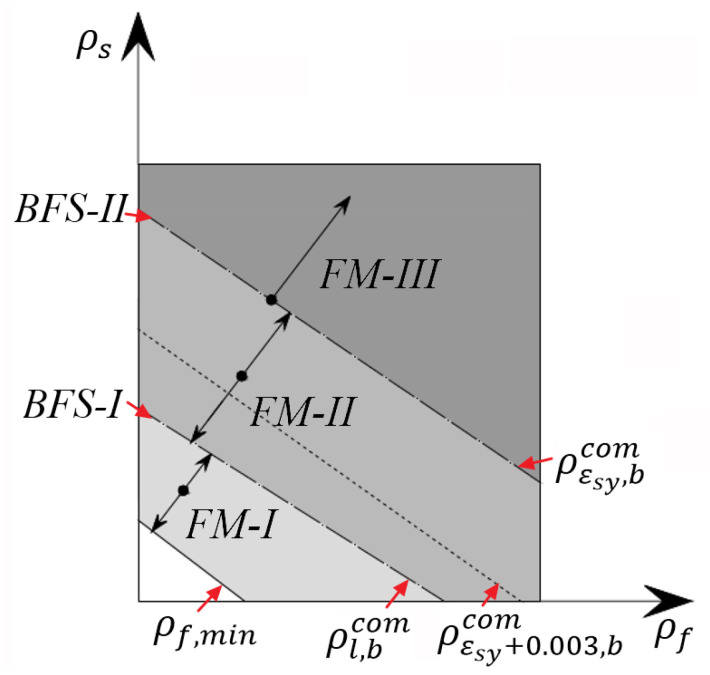
The distribution of reinforcement ratios related with the flexural failure modes.

Finally, the distribution of reinforcement ratios correlated with the three types of flexural failure modes are schematically illustrated in Figure 2.

## 3. Calculation of Flexural Strength

In the proposed design-oriented general calculation approach, the commonly used strain compatibility procedure containing the simplified tensile behavior of steel and FRP reinforcement and the equivalent concrete stress block are assumed to evaluate the nominal flexural strength ($M_{n}$) of hybrid FRP-steel RC beams. Compared with most calculation approaches [1,2,5,6,7,8,9,10,11,13,15,16,17,23,24,25,26] which are merely suitable for the common moderately reinforced beams due to the adopted conventional assumption about the mechanical state of reinforcement ($f_{f}\leq f_{fu},f_{s}=f_{sy}$) and concrete ($\varepsilon_{c}=\varepsilon_{cu}$) at failure, the proposed approach is also applicable to lightly reinforced beams and heavily reinforced beams, which are widely used but rarely studied.

### 3.1. Failure Mode I

At the ultimate state, the steel and FRP reinforcement reach the yield strength and the ultimate tensile strength, respectively; whereas the concrete compressive strain of the extreme compression fiber $\varepsilon_{c}$ is less than $\varepsilon_{cu}$. Hence, the normal Whitney equivalent stress block with two specified parameters ($\alpha_{1}=0.85$, and $\beta_{1}$) [3,25] is not applicable to sectional analysis in this failure mode. To address this issue, Todeschini et al. [3,37,38] presented a similar stress block analytical approach which contains two parameters α and β correlated with the stress-strain relationship of compressive concrete and represented by Equations (14) and (15):

(14) $$\alpha =\frac{0.90ln\left(1+\varepsilon_{c}^{2}/\varepsilon_{c0}^{2}\right)}{\beta \varepsilon_{c}/\varepsilon_{0}}$$

(15) $$\beta =2-\frac{4\left[\varepsilon_{c}/\varepsilon_{c0}-tan^{-1}\left(\varepsilon_{c0}\right)\right]}{\varepsilon_{c}/\varepsilon_{c0}ln\left(1+\varepsilon_{c}^{2}/\varepsilon_{c0}^{2}\right)}$$

where $\varepsilon_{c0}$ is the compressive concrete strain corresponding to maximum strength, computed as $1.71f_{c}^{\prime }/E_{c}$; and $E_{c}$ is the elastic modulus of concrete calculated as 4700$\sqrt{f_{c}^{\prime }}$ in unit of MPa [3,37].

**Figure 3:**
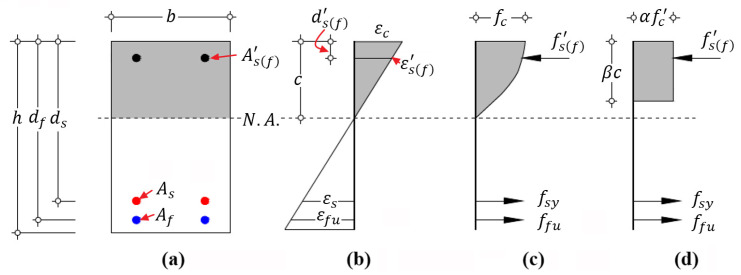
Sectional strain and stress distribution of hybrid RC beam with failure mode I at the ultimate state: (**a**) geometry of cross-section; (**b**) strain distribution; (**c**) stress distribution; and (**d**) equivalent stress distribution.

According to the equilibrium of internal forces on cross-section shown in Figure 3, in which the compressive reinforcement is not considered in this derivation, Equation (16) is obtained:

(16) $$\alpha \beta bcf_{c}^{\prime }=f_{sy}A_{s}+f_{fu}A_{f}$$

where c is the depth of neutral axis measured from the extreme compressive fiber.

Based on the deformation compatibility on cross-section, the relative neutral axis depth $k_{f}$ is shown in Equation (17):

(17) $$k_{f}=\frac{c}{d_{f}}=\frac{\varepsilon_{c}}{\varepsilon_{c}+\varepsilon_{fu}}$$

Rearranging Equation (16), it can also be expressed by Equation (18):

(18) $$k_{f}=\frac{f_{fu}\rho_{l}^{com}}{\alpha \beta f_{c}^{\prime }}$$

To simplify the calculation of nominal flexural strength, a numerical iterative calculation is performed to explore the relationship between the relative mechanical reinforcing index $\rho_{l}^{com}/\rho_{l,b}^{com}$ and the equivalent neutral axis depth $\beta k_{f}$. The detailed calculation procedure is shown as follows:

1. Select a value of mechanical reinforcing index $\rho_{l}^{com}$ in the range of $\rho_{f,min}\leq \rho_{l}^{com}<\rho_{l,b}^{com}$;
2. Assume a value of $\varepsilon_{c}$;
3. Calculate the parameters α and β using Equations (14) and (15);
4. Determine the parameter $k_{f}$ on the basis of Equations (17) and (18), respectively, using the relevant data from steps 2 to 3;
5. Check the values of $k_{f}$ estimated by Equations (17) and (18), respectively. If the values are identical, the relevant data from steps 2 to 4 are the required ones. If not, repeat steps 2 to 5 until they are identical.

**Figure 4:**
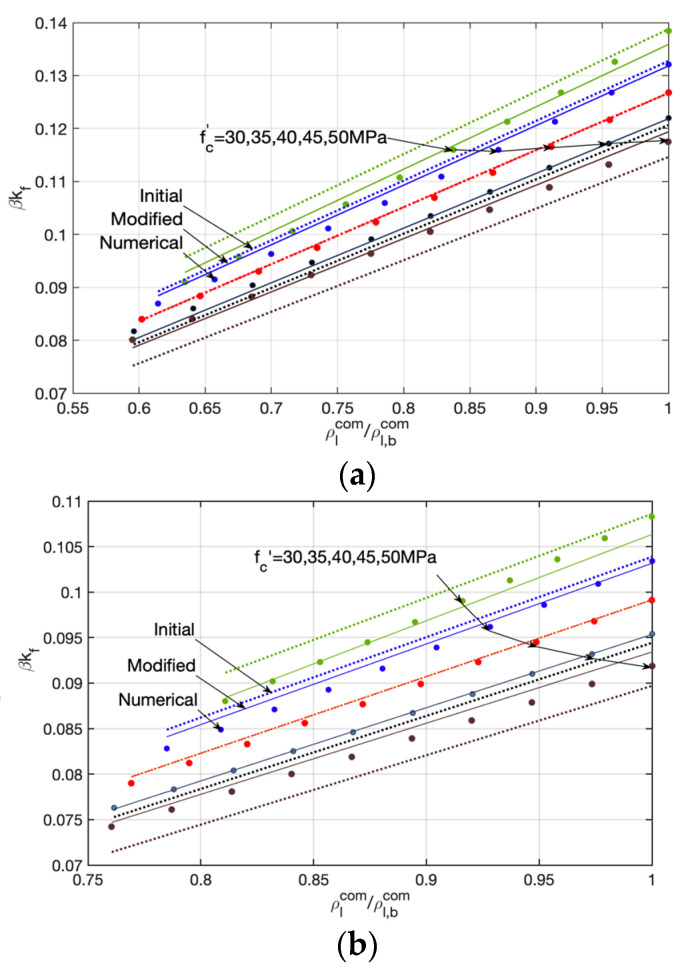

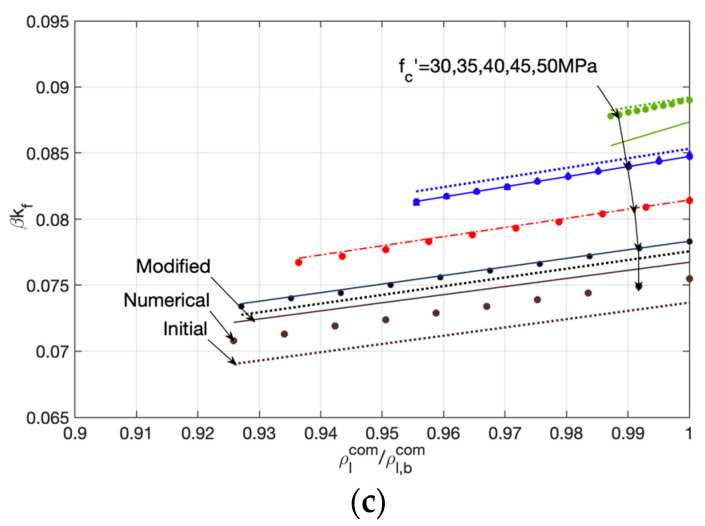
The relationship between equivalent neutral axis depth $\beta k_{f}$ and the relative mechanical reinforcing index $\rho_{l}^{com}/\rho_{l,b}^{com}$. (**a**) $\varepsilon_{fu}=0.15$; (**b**) $\varepsilon_{fu}=0.20$; (**c**) $\varepsilon_{fu}=0.25$.

Subsequently, a parametric analysis following the calculational procedure is implemented and the parameter variables are listed in Table 1. The numerical calculation results indicate that the equivalent neutral axis depth $\beta k_{f}$ possesses an approximately linear correlation with the relative mechanical reinforcing index $\rho_{l}^{com}/\rho_{l,b}^{com}$ [25,38], which are shown by the solid dots (the numerical ones) in Figure 4a–c.

Through a regression analysis, the relationship between the equivalent neutral axis depth $\beta k_{f}$ and the relative mechanical reinforcing index $\rho_{l}^{com}/\rho_{l,b}^{com}$ can be approximately evaluated by Equations (19) and (20), and demonstrated by the dotted lines (the initial ones) in Figure 4a–c.

(19) $$\beta k_{f}=\beta_{1}\left(0.15+0.85\frac{\rho_{l}^{com}}{\rho_{l,b}^{com}}\right)k_{f,b}$$

(20) $$k_{f,b}=\frac{\varepsilon_{cu}}{\varepsilon_{cu}+\varepsilon_{fu}}$$

where $k_{f,b}$ is the relative neutral axis depth in the balanced failure state.

As can be seen from Figure 4a–c, the proposed formula has an excellent prediction for beams with a concrete compressive strength of 40 MPa, but needs further modification for the other concrete compressive strengths. Subsequently, the modified equivalent neutral axis depth ${(\beta k_{f}{)}^{*}}^{}$ expressed by Equations (21) and (22) is suggested.

(21) $${(\beta k_{f}{)}^{*}}^{}=\left(\beta k_{f}-{\left(\beta k_{f}\right)}_{40}\right)\times {\left(0.76/\beta_{1}\right)}^{\frac{f_{c}^{\prime }}{10}}+{\left(\beta k_{f}\right)}_{40};30\;\mathrm{MPa}\leq f_{c}^{\prime }<40\;\mathrm{MPa}$$

(22) $${(\beta k_{f}{)}^{*}}^{}=\left(\beta k_{f}-{\left(\beta k_{f}\right)}_{40}\right)\times {\left(\beta_{1}/0.76\right)}^{\frac{f_{c}^{\prime }}{10}}+{\left(\beta k_{f}\right)}_{40};40\;\mathrm{MPa}\leq f_{c}^{\prime }\leq 50\;\mathrm{MPa}$$

where ${\left(\beta k_{f}\right)}_{40}$ is the equivalent neutral axis depth of the beam with a concrete compressive strength of 40 MPa.

In Figure 4a–c, the modified equations, whose evaluations are demonstrated by the solid lines (the modified ones), show an excellent agreement with the numerical calculation (the numerical ones). The statistical result indicates that the coefficient of determination $R^{2}$ of the proposed modified equations is 99.4%.

Consequently, the nominal flexural strength corresponding to failure mode I can be estimated by Equation (23):

(23) $$M_{n}=f_{fu}A_{f}d_{f}\left(1-\frac{{(\beta k_{f}{)}^{*}}^{}}{2}\right)+f_{sy}A_{s}d_{s}\left(1-\frac{{(\beta k_{f}{)}^{*}}^{}}{2\eta }\right)$$

If the beam is doubly-reinforced, as shown in Figure 3, the strength property of compressive reinforcement is utilized to estimate the upper bound of flexural capacity. Therefore, the mechanical reinforcing index $\rho_{l}^{com}$ considering the compressive steel and FRP reinforcement is expressed by Equations (24) and (25), respectively:

(24) $$\rho_{l}^{com}=\rho_{f}+\rho_{s}\eta \frac{f_{sy}}{f_{fu}}-\rho_{s}^{\prime }\eta \frac{f_{s}^{\prime }}{f_{fu}};f_{s}^{\prime }=min\left(E_{s}\left(\varepsilon_{cu}-\left(\varepsilon_{cu}+\varepsilon_{fu}\right)\frac{d_{s}^{\prime }}{d_{f}}\right),f_{sy}^{\prime }\right)$$

(25) $$\rho_{l}^{com}=\rho_{f}+\rho_{s}\eta \frac{f_{sy}}{f_{fu}}-\rho_{f}^{\prime }\frac{f_{f}^{\prime }}{f_{fu}};f_{f}^{\prime }=min\left(E_{f}\left(\varepsilon_{cu}-\left(\varepsilon_{cu}+\varepsilon_{fu}\right)\frac{d_{f}^{\prime }}{d_{f}}\right),f_{fu}^{\prime }\right)$$

where $f_{s}^{\prime }$ and $f_{f}^{\prime }$ are the compressive stresses of steel and FRP reinforcement, respectively; $f_{sy}^{\prime }$ and $f_{fu}^{\prime }$ are the compressive strengths of steel and FRP reinforcement, respectively; $\rho_{s}^{\prime }$ and $\rho_{f}^{\prime }$ are the ratios of compressive steel and FRP reinforcement, respectively; and $d_{s}^{\prime }$ and $d_{f}^{\prime }$ are the depths of compressive steel and FRP reinforcement measured from the extreme compressive fiber, respectively.

The involvement of compressive reinforcement does not influence the specified linear relationship between equivalent neutral axis depth $\beta k_{f}$ and the relative mechanical reinforcing index $\rho_{l}^{com}/\rho_{l,b}^{com}$, and the modified formulations. Hence, the upper boundary of nominal flexural strength considering steel and FRP compressive reinforcement can be estimated by Equations (26) and (27), respectively:

(26) $$M_{n}=f_{fu}A_{f}d_{f}\left(1-\frac{{(\beta k_{f}{)}^{*}}^{}}{2}\right)+f_{sy}A_{s}d_{s}\left(1-\frac{{(\beta k_{f}{)}^{*}}^{}}{2\eta }\right)+f_{s}^{\prime }A_{s}^{\prime }d_{s}^{\prime }\left(\frac{{(\beta k_{f}{)}^{*}}^{}}{2}\frac{d_{f}}{d_{s}^{\prime }}-1\right)$$

(27) $$M_{n}=f_{fu}A_{f}d_{f}\left(1-\frac{{(\beta k_{f}{)}^{*}}^{}}{2}\right)+f_{sy}A_{s}d_{s}\left(1-\frac{{(\beta k_{f}{)}^{*}}^{}}{2\eta }\right)+f_{f}^{\prime }A_{f}^{\prime }d_{f}^{\prime }\left(\frac{{(\beta k_{f}{)}^{*}}^{}}{2}\frac{d_{f}}{d_{f}^{\prime }}-1\right)$$

Generally, in practical design, the contribution of compressive reinforcement to flexural capacity is neglected in terms of lightly reinforced beams due to its relatively low stress compared with the compressive strength [39].

### 3.2. Failure Mode II

**Figure 5:**
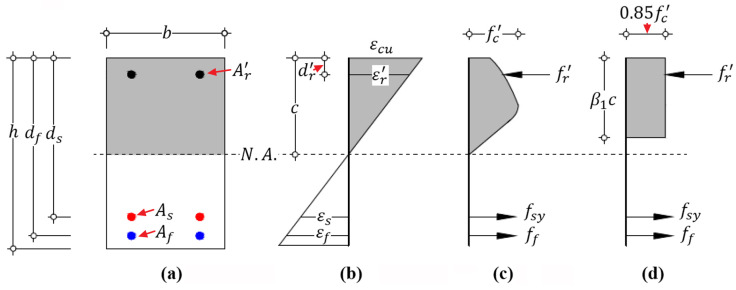
Sectional strain and stress distribution of hybrid RC beam with failure mode II at the ultimate state: (**a**) geometry of cross-section, (**b**) strain distribution, (**c**) stress distribution and (**d**) equivalent stress distribution.

For flexural failure mode II, concrete crushing is caused after steel yielding; the Whitney equivalent stress block can be applied to simplify the calculation of magnitude and location of resultant force in compressive concrete [27,38]; whereas the stress of FRP is unknown. According to the force equilibrium and deformation compatibility on cross-section shown in Figure 5, Equations (28)–(30) can be obtained as:

(28) $$0.85\beta_{1}f_{c}^{\prime }bc+f_{r}^{\prime }A_{r}^{\prime }=f_{sy}A_{s}+f_{f}A_{f}$$

(29) $$f_{r}^{\prime }=E_{r}^{\prime }\frac{c-d_{r}^{\prime }}{c}\varepsilon_{cu}$$

(30) $$f_{f}=E_{f}\frac{d_{f}-c}{c}\varepsilon_{cu}$$

The depth c of neutral axis can be assessed by Equations (31)–(34):

(31) $$c=\frac{-\gamma_{2}+\sqrt{\gamma_{2}^{2}-4\gamma_{1}\gamma_{2}}}{2\gamma_{1}}$$

(32) $$\gamma_{1}=0.85\beta_{1}f_{c}^{\prime }b$$

(33) $$\gamma_{2}=\varepsilon_{cu}\left(E_{r}^{\prime }A_{r}^{\prime }+E_{f}A_{f}\right)-f_{sy}A_{s}$$

(34) $$\gamma_{3}=-\varepsilon_{cu}\left(E_{r}^{\prime }A_{r}^{\prime }d_{r}^{\prime }+E_{f}A_{f}d_{f}\right)$$

where $f_{f}$ is the tensile stress of FRP reinforcement; $f_{r}^{\prime }$ is the stress of compressive reinforcement and is not higher than the compressive strength of FRP or the yield strength of steel; $A_{r}^{\prime }$ is the cross-sectional area of compressive reinforcement; $E_{r}^{\prime }$ is the elastic modulus of compressive reinforcement; $d_{r}^{\prime }$ is the depth of compressive reinforcement; and $\gamma_{1}$, $\gamma_{2}$, and $\gamma_{3}$ are the parameters.

The stress of reinforcement can be estimated by substituting the depth of c into Equations (29) and (30), respectively, and the nominal flexural strength corresponding to failure mode II is estimated by Equation (35):

(35) $$M_{n}=f_{f}A_{f}\left(d_{f}-\frac{\beta_{1}}{2}c\right)+f_{sy}A_{s}\left(d_{s}-\frac{\beta_{1}}{2}c\right)+f_{r}^{\prime }A_{r}^{\prime }\left(\frac{\beta_{1}}{2}c-d_{r}^{\prime }\right)$$

### 3.3. Failure Mode III

**Figure 6:**
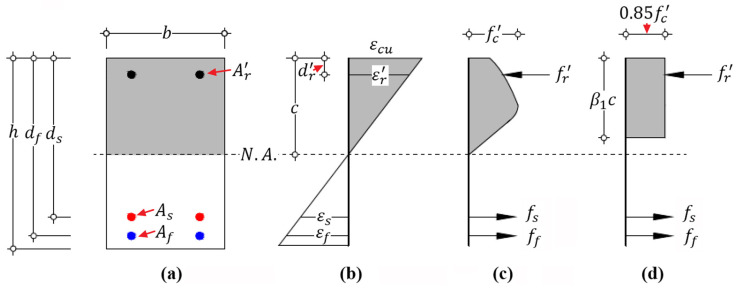
Sectional strain and stress distribution of hybrid RC beam with failure mode III at the ultimate state: (**a**) geometry of cross-section, (**b**) strain distribution, (**c**) stress distribution and (**d**) equivalent stress distribution.

Similar to flexural failure mode II, concrete crushing is caused, and the Whitney equivalent stress block is still applicable to this case; whereas both the steel and FRP reinforcement are in an elastic phase and the stresses are unknown. According to the equilibrium and compatibility conditions on cross-section, illustrated by Figure 6, Equations (36)–(39) are obtained as:

(36) $$0.85\beta_{1}f_{c}^{\prime }bc+f_{r}^{\prime }A_{r}^{\prime }=f_{s}A_{s}+f_{f}A_{f}$$

(37) $$f_{r}^{\prime }=E_{r}^{\prime }\frac{c-d_{r}^{\prime }}{c}\varepsilon_{cu}$$

(38) $$f_{s}=E_{s}\frac{d_{s}-c}{c}\varepsilon_{cu}$$

(39) $$f_{f}=E_{f}\frac{d_{f}-c}{c}\varepsilon_{cu}$$

where $f_{s}$ is the tensile stress of steel reinforcement.

The depth c of neutral axis can be assessed by Equations (40)–(43):

(40) $$c=\frac{-\gamma_{2}+\sqrt{\gamma_{2}^{2}-4\gamma_{1}\gamma_{2}}}{2\gamma_{1}}$$

(41) $$\gamma_{1}=0.85\beta_{1}f_{c}^{\prime }b$$

(42) $$\gamma_{2}=\varepsilon_{cu}\left(E_{r}^{\prime }A_{r}^{\prime }+E_{s}A_{s}+E_{f}A_{f}\right)$$

(43) $$\gamma_{3}=-\varepsilon_{cu}\left(E_{r}^{\prime }A_{r}^{\prime }d_{r}^{\prime }+E_{s}A_{s}d_{s}+E_{f}A_{f}d_{f}\right)$$

Finally, the stresses in reinforcement can be estimated by substituting the depth of c into Equations (37)–(39), respectively, and the nominal flexural strength corresponding to failure mode III can be estimated by Equation (44):

(44) $$M_{n}=f_{f}A_{f}\left(d_{f}-\frac{\beta_{1}}{2}c\right)+f_{s}A_{s}\left(d_{s}-\frac{\beta_{1}}{2}c\right)+f_{r}^{\prime }A_{r}^{\prime }\left(\frac{\beta_{1}}{2}c-d_{r}^{\prime }\right)$$

The beam featured by failure mode III is compression-controlled and fails in a brittle manner by exhibiting small deformation, which is not a favorable design and is often ignored by most calculation approaches [39]. However, it is necessary to calculate the flexural strength of a member with this failure mode, for example, when checking the bearing capacity of existing structures [3].

## 4. Validation of Calculation Approach

To validate the proposed approach, hybrid FRP-steel RC beams experiments found in the literature [1,2,5,7,16,21,23] were collected. All specimens failed in flexure and were characterized by the common flexural failure Modes I and II. Considering the detrimental influence of environmental factors on mechanical properties of FRP, the tensile strength and tensile rupture strain of FRP involved in the evaluation are modified by Equations (45) and (46) [36], respectively:

(45) $$f_{fu}=C_{E}f_{fu}^{*}$$

(46) $$\varepsilon_{fu}=C_{E}\varepsilon_{fu}^{*}$$

where $C_{E}$ is the environmental reduction factor and assigned with 1.0 for carbon FRP (CFRP), 0.9 for glass FRP (GFRP), and 0.8 for aramid FRP (AFRP), respectively; $f_{fu}^{*}$ and $\varepsilon_{fu}^{*}$ are the guaranteed tensile strength and rupture strain of FRP bars, respectively.

In Table 2, the geometrical and modified material properties of specimens are listed, and the comparisons between analytical results and the actual results about the failure modes and the flexural capacity of specimens are reported. The statistical results indicate that the means and standard deviations of the ratio between evaluations and actual results are 0.94 and 12%, respectively, which strongly shows the accuracy and safety of the proposed calculation approach. The existing studies [40,41,42] showed that the reinforcement ratio of FRP significantly influences the stress block parameters which are defined as the function of concrete strength, the sole variable, or constant in the relevant ACI Codes [3,27,33,34,35,36,39] and this study. This ignorance could result in the obvious underestimation in calculations [23,24,40]. Moreover, due to a lack of relevant data about mechanical properties of compressive reinforcement and the flexural experiments of hybrid RC beams featured by the failure mode III, the related calculation formulae need further verifications and improvement.

## 5. Ductility Analysis and Strength Reduction Factor

Currently, there is no design code or guideline to propose direct and detailed suggestions about the strength reduction factor for hybrid FRP-steel RC members, which limits its design and application. Through reference to the latest provisions of the ACI codes about strength reduction factor for steel RC members [34,35], FRP RC members [36], and concrete structures strengthened by externally bonded FRP reinforcement [33] and performing the ductility analysis, a novel strength reduction factor for hybrid FRP-steel RC members is proposed.

### 5.1. Suggestions of Codes and Design Guidelines about Strength Reduction Factor

Concrete members can be defined as tension-controlled and compression-controlled according to load effect. Steel-reinforced concrete beams and slabs are generally designed to a tension-controlled manner demonstrated by steel yielding before concrete crushing. This failure mode associated with steel yielding shows a ductile structural behavior and provides a pronounced warning of member failure. By contrast, compression-controlled steel-reinforced concrete members such as columns, which are more sensitive to variations in concrete strength, exhibit brittle compression failure with little warning [34,35]. For FRP-reinforced concrete members, the compression-controlled behavior featured by concrete crushing prior to FRP rupture is more desirable due to the obvious inelastic response in compressive concrete compared with the brittle FRP rupture [36].

To compensate for the lack of ductility, concrete members should maintain a certain amount of reserve of strength. Consequently, strength reduction factor of ϕ, correlated with ductility (deformability) and safety level, is widely used in practical analysis and design [3,39,43]. In the ACI 318-19 [34], the strength reduction factor is defined according to the net strain $\varepsilon_{st}$ in the extreme tensile layer of reinforcement in a steel RC flexural member. To be specific, the member is defined as tension-controlled if the net tensile strain satisfies the condition of $\varepsilon_{st}\geq \varepsilon_{sy}+0.003$; the corresponding strength reduction factor is assigned with ϕ=0.9. The compression-controlled member is defined as having a net tensile strain $\varepsilon_{st}\leq \varepsilon_{sy}$. Under the balanced failure condition that concrete crushing and steel yielding occur simultaneously, the corresponding strength reduction factor is ϕ=0.65; if the members are spirally reinforced, the strength reduction factor ϕ is assigned with 0.75 due to the higher ductility. Members with net tensile strains between $\varepsilon_{sy}$ and $\varepsilon_{sy}+0.003$ are classified as transition; a linear interpolation of strength reduction factor is defined in this range according to the net tensile strain. The detailed suggestions about strength reduction factor are expressed by Equations (47) and (48), respectively.

(47) $$\phi =\{\begin{array}{c} 0.75\\ 0.75+0.15\frac{\varepsilon_{st}-\varepsilon_{sy}}{0.003}\\ 0.90 \end{array}\begin{array}{c} \varepsilon_{st}\leq \varepsilon_{sy}\\ \varepsilon_{sy}<\varepsilon_{st}<\varepsilon_{sy}+0.003\\ \varepsilon_{st}\geq \varepsilon_{sy}+0.003 \end{array};\mathrm{Spirally reinforced members}$$

(48) $$\phi =\{\begin{array}{c} 0.65\\ 0.65+0.25\frac{\varepsilon_{st}-\varepsilon_{sy}}{0.003}\\ 0.90 \end{array}\begin{array}{c} \varepsilon_{st}\leq \varepsilon_{sy}\\ \varepsilon_{sy}<\varepsilon_{st}<\varepsilon_{sy}+0.003;\\ \varepsilon_{st}\geq \varepsilon_{sy}+0.003 \end{array}\mathrm{Other cases}$$

These provisions do not apply to the lightly reinforced members since whose strains at the extreme compressive fiber of concrete do not reach the ultimate strain of 0.003.

Compared with steel RC members, the FRP RC members show an overall less ductile behavior due to the lack of yielding plateau of FRP reinforcement, which needs to adopt the more stringent strength reduction factor to increase the safety level [3]. The suggestions proposed by ACI 440.1R-15 [36] about the strength reduction factor for FRP RC members are introduced as follows. The balanced failure condition is defined as the simultaneous occurrence of concrete crushing and FRP rupture. If the FRP reinforcement ratio $\rho_{f}$ is not larger than the balanced reinforcement ratio $\rho_{f,b}$, that is, $\rho_{f}\leq \rho_{f,b}$, failure of FRP RC member is induced by rupture of FRP reinforcement; if $\rho_{f}\geq 1.4\rho_{f,b}$, failure is governed by concrete crushing which is the desirable failure mode due to the higher ductility (or deformability). The strength reduction factors corresponding to the two critical states are 0.55 and 0.65, respectively, and there is a linear transition between the two failure modes. The detailed suggestions about strength reduction factor are presented by Equation (49).

(49) $$\phi =\{\begin{array}{c} 0.55\\ 0.30+0.25\frac{\rho_{f}}{\rho_{f,b}}\\ 0.65 \end{array}\;\begin{array}{c} \rho_{f}\leq \rho_{f,b}\\ \rho_{f,b}<\rho_{f}<1.4\rho_{f,b}\\ \rho_{f}\geq 1.4\rho_{f,b} \end{array}\;$$

By contrast, the ACI 440.1R-15 is applicable to lightly reinforced members because the reinforcement ratios $\rho_{f}$ are less than the balanced reinforcement ratio $\rho_{f,b}$.

Hybrid FRP-steel RC members are less ductile than pure steel RC beams, and more ductile than pure FRP RC beams. As concrete structures strengthened by externally bonded FRP reinforcement are considered as a special style of hybrid FRP-steel RC structures, the suggestions proposed by ACI 440.2R-17 [33] about the strength reduction factor served as a good reference in this paper. It follows the design philosophy of ACI 318-19 [34] to relate the strength reduction factor with the net tensile steel strain at nominal strength. Based on the suggestions from ACI 440.2R-17 and ACI 318-19 and ductility analysis, the strength reduction factors of hybrid FRP-steel RC members are assessed and proposed as follows.

### 5.2. Ductility Index

Ductility of conventional steel RC members is defined as the ratio of deformations (curvature, displacement) at the ultimate state to that at steel yielding state [44]. The traditional ductility indices are not applicable to FRP RC members since the members do not show the yielding behavior. Consequently, some deformation-based ductility indices were modified by replacing the deformation at yielding with those corresponding to other characterized stages [45,46]. Meanwhile, the energy-based ductility indices, in which ductility is defined as a capacity for absorbing energy, were proposed to describe the ductility of FRP RC members [10,47,48]. However, the aforementioned indices are not suitable for the hybrid FRP-steel RC members due to the various combinations of the two types of reinforcement with different mechanical properties. To accurately evaluate the ductility of hybrid RC members, Pang et al. [17] proposed a ductility index comprehensively considering the two factors of deformability and energy absorption capacity. The ductility index $\mu_{h}$ is expressed by Equations (50) and (51) [17]:

(50) $$\mu_{h}=\psi D_{u,h}/D_{y,h}$$

(51) $$\psi =U_{H}/U_{S}$$

where ψ is the ductility reduction factor; $D_{u,h}$ is the ultimate curvature of the hybrid RC beam; $D_{y,h}$ is the curvature of the hybrid RC beam at steel yielding; $U_{H}$ is the enclosed area under the moment–curvature curve of the hybrid RC beam; $U_{s}$ is the enclosed area under the moment–curvature curve of the steel RC beam with the equivalent steel area being equal to $A_{s,f}+A_{f}E_{f}/E_{s}$ and other identical configurations such as the effective depth of beam-section; and $\;A_{s,f}$ and $A_{f}$ are the steel and FRP cross-sectional areas in the hybrid RC beam, respectively.

The moment–curvature curves of hybrid RC beam and the counterpart of steel RC beam are simplified as bilinear. Therefore, $U_{H}$ and $U_{s}$ can be calculated by Equations (52) and (53):

(52) $$U_{H}=\frac{M_{y,h}\varphi_{y,h}}{2}+\frac{\left(M_{y,h}+M_{u,h}\right)\left(\varphi_{u,h}-\varphi_{y,h}\right)}{2}$$

(53) $$U_{S}=\frac{M_{y,s}\varphi_{y,s}}{2}+\frac{\left(M_{y,s}+M_{u,s}\right)\left(\varphi_{u,s}-\varphi_{y,s}\right)}{2}$$

where $M_{y,h}$ and $M_{y,s}$ are the yield moments of hybrid RC beam and steel RC beam, respectively; and $\varphi_{y,h}$ and $\varphi_{y,s}$ are the corresponding yield curvatures, respectively.

The moment and corresponding curvature at yielding can be calculated by the linear bending theory [27]. Consequently, the moments $M_{y,h}$ and $M_{y,s}$ are computed by Equations (54)–(59) and Equation (60), respectively:

(54) $$M_{y,h}=A_{s,f}f_{sy}d_{s}\left(1-\frac{k_{s}}{3}\right)+A_{f}f_{f}d_{f}\left(1-\frac{k_{f}}{3}\right)$$

(55) $$k_{s}=\sqrt{{\left(\rho_{s}\beta_{s}+\frac{\rho_{f}\beta_{f}}{\eta }\right)}^{2}\rho_{s}\beta_{s}+2\left(\rho_{s}\beta_{s}+\frac{\rho_{f}\beta_{f}}{\eta^{2}}\right)}-\left(\rho_{s}\beta_{s}+\frac{\rho_{f}\beta_{f}}{\eta }\right)$$

(56) $$k_{f}=\eta k_{s}$$

(57) $$\beta_{s}=\frac{E_{s}}{E_{c}}$$

(58) $$\beta_{f}=\frac{E_{f}}{E_{c}}$$

(59) $$f_{f}=E_{f}\varepsilon_{sy}\frac{\left(1-k_{f}\right)}{\eta \left(1-k_{s}\right)}$$

(60) $$M_{y,s}=A_{s}f_{sy}d_{s}\left(1-\frac{k_{s}}{3}\right)$$

where $k_{s}$ and $k_{f}$ are the relative neutral axis depths in terms of steel and FRP reinforcement, respectively; and $\beta_{s}$ and $\beta_{f}$ are the modulus ratios of steel and FRP to concrete, respectively. Herein moment $M_{y,h}$ is identical with moment $M_{y,s}$.

The yield curvatures $\varphi_{y,h}$ and $\varphi_{y,s}$ are computed by Equation (61):

(61) $$\varphi_{y,h}=\varphi_{y,s}=\frac{\varepsilon_{sy}}{\left(1-k_{s}\right)d_{s}}$$

The ultimate moments $M_{u,h}$ of hybrid RC beam and $M_{u,s}$ of steel RC beam can be computed by Equations (62) and (63), respectively, derived from Equation (35).

(62) $$M_{u,h}=A_{s,f}f_{sy}\left(d_{s}-\frac{\beta_{1}}{2}c\right)+A_{f}f_{f}\left(d_{f}-\frac{\beta_{1}}{2}c\right)$$

(63) $$M_{u,s}=A_{s}f_{sy}\left(d_{s}-\frac{\beta_{1}}{2}c\right)$$

If the calculated ultimate moments $M_{u,h}$ and $M_{u,s}$ are less than the yield moments $M_{y,h}$ and $M_{y,s}$, as in cases with a large percentage of tensile reinforcement, the yield moments should be taken as equal to the ultimate moments [27]. The ultimate curvatures $\varphi_{u,h}$ and $\varphi_{u,s}$ are calculated by Equation (64):

(64) $$\varphi_{u,h},\;\varphi_{u,s}=\frac{\varepsilon_{cu}}{c}=\frac{\varepsilon_{cu}+\varepsilon_{st}}{d_{s}}$$

The introduced ductility index and the extended formulae are employed to assess the ductility level of hybrid FRP-steel RC members.

### 5.3. Ductility Level and Strength Reduction Factor

In the ductility analysis of conventional steel RC structures, both the net tensile steel strain at nominal strength and reinforcement ratio can be employed to describe ductility level, but the net tensile steel strain is more desirable for simplicity [27,39,48,49]. This philosophy has been adopted by ACI 318-19 [34] and ACI 440.2R-17 [33] to define the strength reduction factor. Equations (8) and (9) demonstrate that the net tensile steel strain relates to steel and FRP reinforcement ratio of hybrid RC beams. Thus, the variation of ductility level of hybrid RC beams can be evaluated by assigning the net tensile strain $\varepsilon_{st}$ with the values in the defined range. It should be pointed out that this analytical strategy is only applicable to and required for the hybrid RC beams featured by failure mode II. On one hand, in terms of the beam with failure mode III, it is heavily (over)-reinforced and compression-controlled due to the obvious compressive failure feature in concrete and the elastic feature in tensile reinforcement. Therefore, its ductility level is obviously lower than that under the balanced failure state II. On the other hand, for the hybrid RC beam with failure mode I, its ductility level is governed by both the rupture strain of FRP and concrete compressive strain, so the reinforcement ratio is the preferred parameter for defining the ductility level.

**Figure 7:**
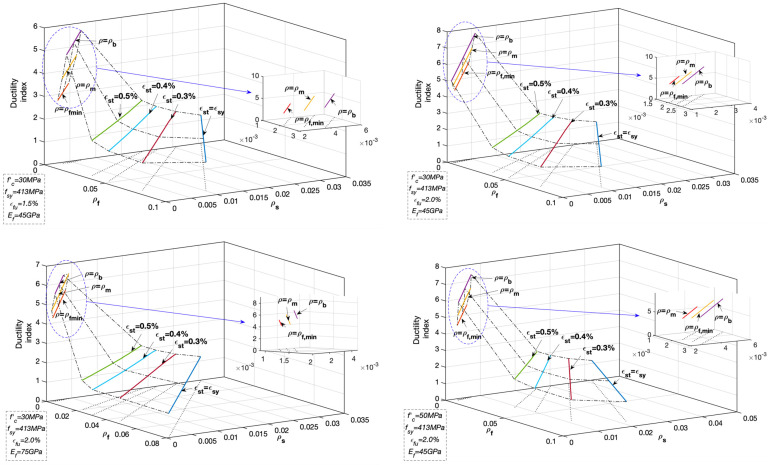
Relationship between ductility level and net tensile steel strain and reinforcement ratio (Note: $\rho_{m}=0.5\left(\rho_{f,min}+\rho_{l,b}^{com}\right)$; and $\rho_{b}=\rho_{l,b}^{com}$ ).

Subsequently, a parametric analysis is performed to investigate the ductility level of hybrid RC beams with varied geometrical and mechanical properties. The investigated hybrid RC beams contain the failure modes I and II. Specifically, for the beam featured by failure mode I, the ductility levels corresponding to reinforcement ratio $\rho_{l}^{com}$ being equal to $\rho_{f,min}$, 0.5$\left(\rho_{f,min}+\rho_{l,b}^{com}\right)$, and $\rho_{l,b}^{com}$ were assessed, respectively; for the beam featured by failure mode II, the ductility levels corresponding to net tensile steel strain of $\varepsilon_{sy}$, 0.003, 0.004 and 0.005 ($\varepsilon_{sy}+0.003$) were evaluated, respectively. The critical net tensile steel strain of 0.005 defined in the ACI 318-14 [35] and ACI 440.2R-17 [33] was replaced by $\varepsilon_{sy}+0.003$ in the latest ACI 318-19 [34] to accommodate the steel reinforcement of higher grades, which does not influence the results of parametric analysis. The other variables are listed in Table 1. The evaluation results about the relationship between ductility level and the variables of net tensile steel strain and reinforcement ratio are shown in Figure 7.

As shown in Figure 7, in terms of the hybrid RC beams with failure mode II, the ductility level gradually increases as net tensile steel strain increases. The monotonic increasing trend continues until the balanced failure state I is reached. Then the ductility level gradually reduces as the reinforcement ratio decreases in terms of the beams with failure mode I. This variation feature of ductility can be illustrated by the deformation ingredient of the hybrid RC beams with varied reinforcement ratios. For a lightly reinforced concrete beam, the contribution to flexural deformation from the tensile zone is larger than that from compressive concrete which is not crushed at failure [28]; for a heavily-reinforced concrete beam, the main contribution comes from the compressive zone due to concrete crushing, and the tensile zone plays the minor role in flexural deformation due to the large reinforcement ratio; while for the moderately-reinforced concrete beam, both tensile and compressive zones create the obvious deformation and induce the large flexural deformation [12,50].

As the net tensile steel strain increases from $\varepsilon_{sy}$ to 0.005 ($\varepsilon_{sy}+0.003$), the variation trend of ductility of the hybrid RC beam with failure mode II well coincides with the linear increase in strength reduction factor from 0.65 to 0.90 defined for conventional steel RC beams and the strengthened concrete beams using externally bonded FRP reinforcement. It proves that the net tensile steel strain in hybrid RC beam could reflect the ductility level to represent the strength reduction factor similar to conventional steel RC beams and the strengthened concrete beams with FRP reinforcement. Hence, the identical provisions from ACI 318-19 and ACI 440.2R-17 about the strength reduction factor are suggested to be used for the hybrid RC beams. When net tensile steel strain is greater than 0.005 ($\varepsilon_{sy}+0.003$), the ductility level becomes higher and it is conservatively safe to adopt the constant strength reduction factor of 0.90. In the range of $\varepsilon_{st}<\varepsilon_{sy}$, the constant strength reduction factor is assigned with 0.65.

The lightly reinforced hybrid concrete beam with failure mode I is featured by FRP rupture and steel yielding. If the strength reduction factor is roughly assigned with 0.55, which is defined for the pure FRP RC members, the ductility level of hybrid FRP-steel RC beam is generally underestimated without considering the contribution of steel reinforcement to improving ductility. Thus, it is suggested that the strength reduction factors corresponding to the minimum reinforcement ratio $\rho_{f,min}$ and to the balanced reinforcement ratio $\rho_{l,b}^{com}$ are assigned with 0.55 and 0.9, respectively; and a linear transition for the reduction factor is suggested between the two critical states [43].

**Figure 8:**
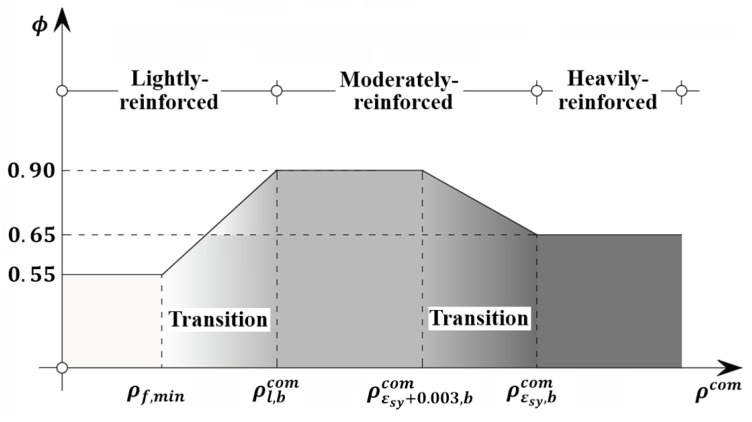
Proposed strength reduction factor.

Subsequently, the global relationship between strength reduction factor and reinforcement ratio and net tensile steel strain is presented in Equation (65) and shown in Figure 8.

(65) $$\phi =\{\begin{array}{cc} 0.55, & \rho_{l}^{com}\leq \rho_{f,min}\\ 0.55+0.35\frac{\rho_{l}^{com}-\rho_{f,min}}{\rho_{l,b}^{com}-\rho_{f,min}}, & \rho_{f,min}<\rho_{l}^{com}<\rho_{l,b}^{com}\\ 0.90, & \rho_{l}^{com}\geq \rho_{l,b}^{com},\;\rho_{\varepsilon_{sy+0.003}}^{com}\leq \rho_{\varepsilon_{sy+0.003,b}}^{com}\\ 0.65+0.25\frac{\left(\varepsilon_{st}-\varepsilon_{sy}\right)}{0.003}, & \varepsilon_{sy}<\varepsilon_{st}<\varepsilon_{sy}+0.003\;\left(\rho_{\varepsilon_{sy+0.003}}^{com}>\rho_{\varepsilon_{sy+0.003,b}}^{com},\;\rho_{\varepsilon_{sy}}^{com}<\rho_{\varepsilon_{sy},b}^{com}\right)\\ 0.65, & \rho_{\varepsilon_{sy}}^{com}\geq \rho_{\varepsilon_{sy},b}^{com} \end{array}$$

**Figure 9:**
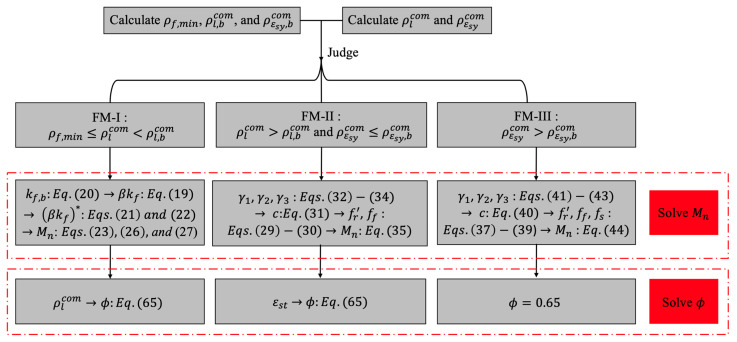
Design flow chart to estimate the factored bending moment capacity.

Finally, a design flow chart to estimate the factored bending moment capacity of $\phi M_{n}$ for each flexural failure mode is illustrated by Figure 9.

Following the relevant provisions of ACI codes for steel-reinforced [34,35] and FRP-reinforced members [36], the presented strength reduction factor for hybrid FRP-steel RC beams can be also applicable to the hybrid FRP-steel concrete members subjected to uniaxial compression, tension, and the combined moment and axial force, and characterized by other relevant failure modes.

## 6. Conclusions

In this paper, a methodology for the flexural strength design of hybrid FRP-steel reinforced concrete beams was proposed. Specifically, the mechanical features of reinforcement and concrete and the ranges of reinforcement ratio corresponding to lightly-reinforced, moderately-reinforced, and heavily-reinforced hybrid FRP-steel RC beams were analyzed. Subsequently, a simplified and straightforward analytical procedure to evaluate the nominal flexural strength of hybrid FRP-steel RC beams with common flexural failure modes was established. Finally, the novel relationship between strength reduction factor and reinforcement ratio and net tensile steel strain was proposed based on ductility analysis, offering instructive suggestions for the practical design.

The conclusions of this study can be drawn as follows:

1. The design-oriented allowable ranges of reinforcement ratio corresponding to three common flexural failure modes of hybrid FRP-steel RC beams were specified according to the mechanical features of reinforcement and concrete and the latest codified provisions of longitudinal reinforcement conditions to guarantee the sufficient ductility level. For beams featured by the preferable flexural failure mode, the detailed relationship between net tensile steel strain level and reinforcement ratio was established to evaluate sectional ductility;
2. The general calculation approach of nominal flexural strength was proposed for hybrid FRP-steel RC beams. In addition to the common moderately-reinforced beams, the approach was also applicable to lightly-reinforced beams and heavily-reinforced beams, which are widely used but rarely studied. Furthermore, the calculation process was simplified using the derived relationship between relative neutral axis depth and the reinforcement ratio, and the calculation accuracy was successfully validated by the experimental results. However, the stress block parameters are expected to be modified to consider the effect of FRP reinforcement ratio, and the proposed formulae for lightly-reinforced and heavily-reinforced beams need further verification and improvement due to rare experimental results;
3. For hybrid FRP-steel RC beams featured by flexural failure modes II and III (i.e., moderately- and heavily-reinforced beams), the strength reduction factor can be used as that defined for conventional steel RC beams. For hybrid FRP-steel RC beam with failure mode I (i.e., lightly-reinforced concrete beams), the strength reduction factor was proposed in terms of different reinforcement ratios. It can be adopted as 0.55 and 0.9 for beams with the minimum reinforcement ratio $\rho_{f,min}$ and the balanced reinforcement ratio $\rho_{l,b}^{com}$, respectively; and a linear transition for the strength reduction factor is assumed between the two critical reinforcement ratios;
4. The proposed design methodology, based on the design philosophy and provisions of the relevant ACI codes, can be further modified and extended according to other design standards of practice.

## Figures and Tables

**Table 1:** Parameter variables.

Parameter	Value
Yield strength of steel $f_{y}$	413 and 550 MPa
Ultimate tensile strain of FRP $\varepsilon_{fu}$	0.015, 0.02 and 0.025
Compressive strength of concrete $f_{c}^{\prime }$	30, 35, 40, 45 and 50 MPa
Modulus of elasticity of FRP $E_{f}$	45, 75 and 145 GPa

**Table 2:** Comparison between the analytical results and experimental results of hybrid RC beams.

Reference	Specimen	Geometries		$f_{c}^{\prime }\;(\mathrm{MPa})$	Steel		FRP			$\rho_{l}^{com}$	$\rho_{l,b}^{com}$	$\rho_{\varepsilon_{sy}}^{com}$	$\rho_{\varepsilon_{sy},b}^{com}$	Actual Mode	Analytical Mode	$M_{a}$ (kN·m)	$\frac{M_{a}}{M_{e}}$
		*b* (mm)	*h* (mm)		$A_{s}\;({\mathrm{mm}}^{2})$	$f_{y}\;(\mathrm{MPa})$	$A_{f}\;({\mathrm{mm}}^{2})$	$f_{fu}\;(\mathrm{MPa})$	$E_{f}\;(\mathrm{GPa})$								
Aiello et al. [1]	A1	150	200	45.7	100.48	465	88.31	1674	49	7.11	2.58	2.60	15.15	FM-II	FM-II	21.10	0.84
	A2	150	200	45.7	100.48	465	157	1366	50.1	9.39	3.13	3.02	14.86	FM-II	FM-II	25.64	0.90
	A3	150	200	45.7	226.08	465	235.5	1366	50.1	15.63	3.16	6.09	15.07	FM-II	FM-II	32.09	0.90
	C1	150	200	45.7	100.48	465	88.31	1674	49	6.85	2.48	2.16	15.73	FM-II	FM-II	22.27	0.89
Leung et al. [2]	L2	150	200	28.5	157	460	142.6	760	40.8	8.13	3.09	4.52	11.09	FM-II	FM-II	15.58	0.70
	L5	150	200	28.5	157	460	213.9	760	40.8	10.38	3.03	4.94	10.90	FM-II	FM-II	17.42	0.76
	H2	150	200	48.8	157	460	142.6	760	40.8	8.13	4.33	4.52	15.56	FM-II	FM-II	18.76	0.89
	H5	150	200	48.8	157	460	213.9	760	40.8	10.38	4.25	4.94	15.30	FM-II	FM-II	21.33	0.79
Qu et al. [5]	B3	180	250	28.1	226.08	363	253.23	782	45	6.07	3.60	2.59	12.65	FM-II	FM-II	37.86	0.99
	B4	180	250	28.1	200.96	336	396.91	755	41	7.76	3.43	2.40	13.01	FM-II	FM-II	40.67	1.03
	B5	180	250	29.2	401.92	336	141.69	778	37.7	5.64	3.24	3.64	13.52	FM-II	FM-II	37.69	1.04
	B6	180	250	29.2	401.92	336	253.23	782	45	7.41	3.74	3.89	13.52	FM-II	FM-II	44.14	1.04
	B7	180	250	34.6	113.04	363	141.69	778	37.7	3.26	3.46	1.28	14.02	FM-I	FM-I	26.79	1.14
	B8	180	250	34.6	1205.76	336	396.91	755	41	17.81	4.35	12.82	14.86	FM-II	FM-II	68.52	1.08
Safan [7]	B10/6S	100	200	30	157	530	56.6	780	41	7.96	4.21	6.64	10.89	FM-II	FM-II	12.66	0.90
	B10/8S	100	200	30	157	530	100.6	755	39	9.37	3.99	7.01	10.71	FM-II	FM-II	13.90	0.96
	B12/6S	100	200	30	226	470	56.6	780	41	9.70	4.29	8.35	11.60	FM-II	FM-II	14.13	0.95
	B12/8S	100	200	30	226	470	100.6	755	39	11.08	4.07	8.69	11.43	FM-II	FM-II	15.12	0.93
Araba et al. [16]	SH1	200	300	53.72	100.48	580	169.81	1100	45.69	4.14	5.02	2.07	14.06	FM-I	FM-I	49.32	0.80
	SH2	200	300	56.61	401.92	580	278.97	1200	55	10.46	5.05	6.70	15.09	FM-II	FM-II	81.57	0.74
Lau et al. [21]	G03MD1	280	380	41.3	981.7	336	283.5	588	39.5	4.86	5.34	3.67	16.89	FM-I	FM-I	146.57	1.00
	G10T07	280	380	39.8	628.3	597	981.7	582	38	8.73	5.05	5.07	12.94	FM-II	FM-II	205.09	0.95
	G06T1	280	380	44.6	981.7	550	567.1	588	39.5	8.46	5.58	6.33	14.36	FM-II	FM-II	220.43	0.97
Ruan et al. [23]	2G12-2S12	180	300	30.32	226.2	517	226.2	868.22	40.06	5.77	3.16	2.95	11.49	FM-II	FM-II	54.84	0.95
	2G16-2S12	180	300	30.32	226.2	517	402.1	958.2	45.69	9.00	3.24	3.45	11.52	FM-II	FM-II	65.98	1.04
	2G12-1S16	180	300	30.32	201.1	540	226.2	868.22	40.06	5.61	3.17	2.83	11.25	FM-II	FM-II	53.54	0.95
	2G16-1S16	180	300	30.32	201.1	540	402.1	958.2	45.69	8.84	3.25	3.35	11.26	FM-II	FM-II	64.82	0.97
	2G12-2S12(D)	180	300	30.32	226.2	517	226.2	868.22	40.06	6.13	3.36	3.52	11.11	FM-II	FM-II	50.51	0.94
	2G16-2S12(D)	180	300	30.32	226.2	517	402.1	958.2	45.69	9.40	3.38	4.28	10.82	FM-II	FM-II	61.18	1.21
Average	-	-	-	-	-	-	-	-	-	-	-	-	-	-	-	-	0.94
Standard deviation	-	-	-	-	-	-	-	-	-	-	-	-	-	-	-	-	12%

Note: actual mode = actual flexural failure mode; analytical mode = predicted flexural failure mode; FM-I = failure mode I; FM-II = failure mode II; $M_{a}$ = analytical moment capacity; and $M_{e}$ = experimental moment capacity.

## Data Availability

No new data were created or analyzed in this study. Data sharing is not applicable to this article.
